# Spatial and temporal assessment of heavy metal contamination in urban soils of Mosul post war (2022–2023): environmental implications and risk evaluation

**DOI:** 10.1007/s11356-026-37536-y

**Published:** 2026-03-06

**Authors:** Zena Altahaan, Daniel Dobslaw

**Affiliations:** 1 Institute of Sanitary Engineering, Water Quality and Solid Waste Management, University of Stuttgart, 70569 Stuttgart, Germany; 2 Institute of Spatial and Regional Planning, University of Stuttgart, 70569 Stuttgart, Germany

**Keywords:** Soil pollution, Heavy metals, Seasonal variation, Annual variation, Post-conflict environment, Mosul City

## Abstract

Soil contamination with heavy metals remains a major environmental concern in post-conflict Mosul City, posing risks to agriculture, ecosystems, and human health. This study investigated seasonal and interannual variations in cadmium (Cd), lead (Pb), zinc (Zn), chromium (Cr), and nickel (Ni), along with key soil properties (pH, electrical conductivity, and salinity), across urban soils during 2022–2023 by applying multivariate statistics and ArcGIS-based spatial analysis. Severe contamination was identified in 39.47% of the study area (S3, S4, and S7), while high contamination affected an additional 40.09% (S6 and S8); the remaining 20.43% (S1, S2, and S5) showed moderate contamination. Soil pH remained relatively stable, whereas electrical conductivity, salinity, and heavy metals particularly Pb, Zn, Cr, and Ni exhibited higher variability. Strong inter-metal correlations and principal component analysis indicated dominant anthropogenic influences, with heavy metals forming one principal component and EC–salinity another. Seasonal variations were evident in 2022, most likely associated with rainfall-driven leaching, but were negligible in 2023 under drier conditions. Spatial patterns reflected the combined influence of soil properties, climatic conditions, and human activities. These findings highlight the persistence of soil contamination in post-conflict Mosul and underscore the need for immediate remediation in high-risk zones, continued monitoring in moderately contaminated areas, and adaptive land management practices to reduce environmental and health risks. The integrated multivariate–spatial framework offers a transferable approach for assessing soil contamination in conflict-affected urban environments.

## Introduction

Soil contamination by heavy metals is a major environmental issue affecting ecosystem functioning, agricultural productivity, and public health (Smith 2023). In post-conflict cities such as Mosul, Iraq, this problem is further intensified by infrastructure destruction and insufficient environmental management following the armed conflict. During 2013–2017, intensive military activities, including the bombing of industrial facilities and the ignition of oil wells and sulfur storage sites, contributed to widespread environmental degradation and the potential accumulation of toxic metals in urban soils (UNEP 2017; UNEP and OCHA 2016; UN-Habitat 2016). Previous evidence indicates elevated levels of metals such as Cd and Pb in and around conflict-affected zones (Altahaan & Dobslaw 2024c; Mohammed et al. 2020).

Metal concentrations in soils may vary spatially and temporally due to both climate and human activities**.** Seasonal variations in rainfall and evaporation can influence metal mobility through leaching processes and surface concentration effects, while land-use change, industrial emissions, and traffic-related sources may sustain long-term enrichment (Alloway 2013; Kabata-Pendias and Mukherjee 2007; Chen et al. 2015a, b; Li et al. 2014). Understanding both seasonal and interannual variability is therefore essential for effective monitoring and prioritization of remediation strategies in recovering urban environments.

This study investigates the seasonal and interannual variability of Cd, Pb, Zn, Cr, and Ni in Mosul urban soils during 2022–2023. Ninety-six samples were collected from eight sites representing both conflict-impacted areas and adjacent zones. Temporal changes were evaluated using *t*-tests and Spearman correlation; spatial patterns were mapped in ArcGIS, variability was assessed using the coefficient of variation (CV), and potential sources were explored using principal component analysis (PCA).

The present study was conducted to test the following hypotheses:

i. Spatial variability influences the distribution of heavy metals in soil across the study area.
ii. Seasonal meteorological conditions, particularly variations in rainfall and temperature, affect soil physicochemical properties and heavy metal concentrations.
iii. Over time, natural processes contribute to changes in heavy metal behavior and persistence in soil, influencing their mobility and potential attenuation in post-conflict environments.

### Previous study

Numerous studies have examined the impact of armed conflicts on soil contamination in Iraq and other conflict-affected regions, highlighting elevated concentrations of heavy metals and associated ecological and health risks.

In Mosul, Abdulsattar (2018) investigated soil pollution in the Danfeli stream valley and found extremely high levels of cadmium (Cd), substantial contamination with lead (Pb), and moderate levels of nickel (Ni), suggesting that war-related activities were the dominant source of pollution. Similarly, Hamad et al. (2019) assessed heavy metal contamination in Halgurd-Sakran National Park following controlled mine detonations during demining operations. The study reported high to moderate contamination with copper (Cu), lead (Pb), zinc (Zn), nickel (Ni), cobalt (Co), and manganese (Mn), as measured by the geo-accumulation index (Igeo).

Znad & Fadhel (2020) explored seasonal variations in soil contamination in the Al-Karama industrial zone of western Mosul. Using X-ray fluorescence, they found elevated concentrations of selenium (Se), manganese (Mn), mercury (Hg), cobalt (Co), and antimony (Sb), with industrial emissions identified as the primary source. In another study conducted shortly after the war, Mohammed et al. (2020) analyzed soil samples from the old city of Mosul and detected significantly elevated levels of Cd, Ni, Cr, Zn, and Cu, with marked differences between soil layers. The *t*-test results supported the hypothesis that heavy metal accumulation was primarily linked to war-related disturbances.

Beyond Mosul, (Al Lami et al. 2019) reported exceptionally high levels of trace metals including arsenic (As), silver (Ag), vanadium (V), cesium (Cs), tellurium (Te), titanium (Ti), and iron (Fe) in the soils of Al-Anbar City, western Iraq, following the ISIS conflict. These findings further confirm that warfare activities introduce a wide range of contaminants into urban soils.

The relevance of seasonal dynamics in heavy metal contamination has also been demonstrated in international case studies. It emphasized the importance of understanding spatial and temporal variability in soil pollution for designing effective remediation plans. Chang and Kim (2012) highlighted how fluctuations in temperature and precipitation influence contaminant mobility and bioavailability. For example, Oluyemi found that heavy metal concentrations in soils and crops near a Nigerian landfill were consistently higher during the dry season. Their study reported critical levels of As, Cd, Cr, Ni, and Pb in edible plant tissues, emphasizing the food safety implications of seasonal pollution cycles.

Similarly, (Thakkar et al. 2024)al.2024 investigated the seasonal distribution of 21 heavy metals in soils between the Mahi and Dhadhar rivers in Gujarat, India. The study identified high contamination during the monsoon and post-monsoon periods, particularly from metals such as Ag, Cd, Cr, Cu, Co, Ni, and Pb, which posed significant ecological and carcinogenic risks. (Dhaliwal et al. 2021) also assessed the health risks associated with heavy metal exposure in Punjab, India, using a multi-index approach. Their findings highlighted Ni as the most critical contaminant based on its bioaccumulation in crops and potential to affect human health.

Collectively, these studies underscore the need for long-term monitoring and remediation programs in post-conflict areas, with a special focus on seasonal patterns, pollutant mobility, and human exposure risks. They also affirm the value of combining geochemical analysis with statistical tools to assess contamination dynamics more effectively.

## Materials and methods

### Study area

The study was conducted within the urban boundaries of Mosul, located in northwestern Iraq, spanning the geographical coordinates of 35°–37° N latitude and 41°–44° E longitude. According to the World Reference Base for Soil Resources (WRB 2022), the soils in this region are diverse and classified into several types based on their physicochemical characteristics within the topsoil layer (0–15 cm). All soil samples were collected at a consistent depth. Sampling was conducted during daytime hours under comparable field conditions to minimize temporal variability. The main soil categories include:

- **Calcisols**: Rich in calcium carbonate, commonly found in arid and semi-arid climates.
- **Gypsisols**: Characterized by elevated gypsum content, also prevalent in dry environments.
- **Vertisols**: Clay-dominated soils known for their shrink-swell behavior with changes in moisture.
- **Fluvisols**: Typically located in floodplain areas and influenced by fluvial sedimentation.

**Fig. 1:**
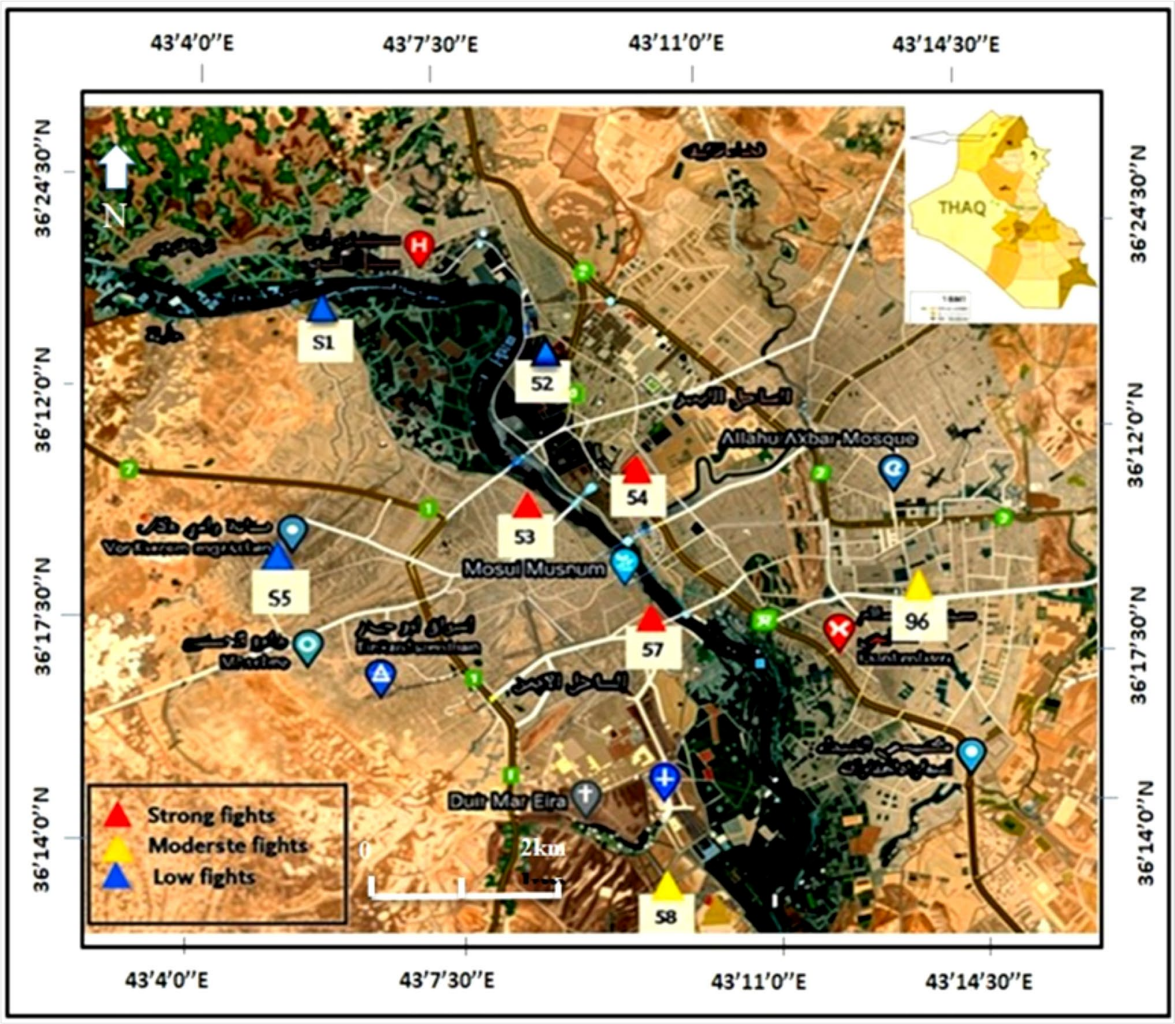
Sampling sites map

The particle size distribution and soil texture vary considerably across sampling sites due to differing environmental influences and land uses. Sampling locations were determined based on spatial heterogeneity and reference to prior geospatial assessments (Al-Taie et al. 2015), and are presented in Fig. 1.

For each site, a 20 × 20 m sampling grid was established. Soil samples were collected from a depth of 0–15 cm at 10-m intervals along the grid axes, resulting in 12 subsamples per location and sampling round. These were homogenized to produce one representative composite sample per site per season. A total of eight locations were selected across the city, encompassing areas with varying degrees of war-related disturbance.

Sampling was performed during four distinct campaigns: winter (January–March 2022), summer (July–September 2022), winter (January–March 2023), and summer (July–September 2023), representing both wet and dry climatic conditions. Across all campaigns, 96 composite samples were obtained. Samples were stored in 250 mL gas-tight polyethylene bags, immediately cooled to − 4 °C, and maintained under refrigeration until laboratory analysis. Recorded soil surface temperatures ranged from 0 to 10 °C in the cooler seasons (series 1 and 3) and 32–45 °C during the hotter periods (series 2 and 4).

Site selection considered both proximity to former conflict zones and logistical accessibility. Three locations (S3, S4, S7) were situated near heavily impacted areas, while five others (S1, S2, S5, S6, S8) served as control or adjacent zones, see Table 1. All samples were analyzed in the environmental laboratory using standardized procedures. Measured parameters included soil pH, electrical conductivity (EC), salinity, and concentrations of cadmium (Cd), lead (Pb), zinc (Zn), chromium (Cr), and nickel (Ni), determined via atomic absorption spectroscopy (AAS).

**Table 1:** Location of soil sampling sites in Mosul city

Site no	Location	Latitude (N)	Longitude (E)	Site description	Soil description	Soil type(s)	No. of samples
S1	Al-Rashidia	36.40005	43.103287	Residential area	Clayey and sandy soil with some organic matter and minerals such as quartz and feldspar	Calcisols, Vertisols, Fluvisols	96
S2	Mosul Forest	36.383410	43.117947	Recreational area	—	—	96
S3	Old City	36.34022	43.128143	Commercial & residential area	Mixture of clay and sand with a higher percentage of organic matter; intensively mixed due to explosion impacts	Calcisols, Vertisols, Gypsisols	96
S4	Al-Faysalia	36.34651	43.142455	Residential area	—	Fluvisols	96
S5	Al-Yarmuk Flats	36.321480	43.076216	Residential area	Mixture of clay and sand with a high percentage of organic matter; contains minerals such as illite and kaolinite	Calcisols, Vertisols, Gypsisols	96
S6	Al-Karama Industrial Area	36.34201	43.205897	Industrial area	—	Fluvisols	96
S7	Al-Danadan	36.32333	43.161559	Residential area	Heavy clay with a high organic matter content; affected by explosion-induced mixing; contains vermiculite and mica minerals	Calcisols, Vertisols, Fluvisols	96
S8	Al-Busaf	36.27324	43.163325	Agricultural area			96

### Measurement methods

To evaluate the physical and chemical characteristics of the soil samples, standard laboratory protocols were applied. The following parameters were measured: temperature, pH, electrical conductivity (EC), salinity, and the concentrations of five heavy metals—cadmium (Cd), lead (Pb), zinc (Zn), chromium (Cr), and nickel (Ni).

#### Physiochemical parameters

A subsample of 10–15 g of air-dried soil was thoroughly mixed with 25 mL of distilled water. After 2–3 min of continuous stirring, the suspension was allowed to stabilize, and the supernatant was analyzed for pH, electrical conductivity (EC), and salinity. These parameters were initially measured using a portable multiparameter water quality tester (Oumefar 5-in-1, UPC 886108495111). The portable multiparameter instrument was calibrated according to the manufacturer’s instructions prior to each sampling campaign using appropriate standard solutions. Calibration was verified in the field at six-month intervals to ensure measurement accuracy. Soil temperature was recorded directly in the field at the time of sampling to account for seasonal and site-specific thermal variations.

#### Heavy metal

Soil samples were oven-dried at 104 °C for 48 h, ground to a fine powder using a mortar and pestle, and sieved through a 106 µm stainless steel mesh. The prepared samples were stored in polyethylene containers prior to digestion.

The digestion procedure followed the classical wet digestion method described by Jackson (1958). Approximately 0.5 g of dried and homogenized soil was transferred to a digestion flask and mixed with 5 mL of an acid mixture comprising concentrated sulfuric acid (H₂SO₄, 96%), nitric acid (HNO₃, 68%), and perchloric acid (HClO₄, 70%) in a volume ratio of 3:1:1. The mixture was heated at 90 °C for 2 h under a fume hood until complete digestion was achieved. The digested solution was then diluted to a final volume of 25 mL with deionized water. Soil samples were digested following the method described by Jackson (1958), which is widely used for total metal extraction in soil studies. This method was selected due to its effectiveness in dissolving metal-bearing soil phases and its frequent application in regional and comparative environmental assessments. The approach provides results comparable to EPA 3050B-type digestion methods for evaluating total metal concentrations in soils.

The concentration of heavy metals in the digested solutions was determined using Atomic Absorption Spectroscopy (AAS) following the methods established by APHA, AWWA, WEF (2017). Calibration curves were generated using certified standard solutions, and a regression equation was applied to convert absorbance values into concentrations expressed in parts per million (mg/kg), in accordance with protocols outlined by Haswell (1991) and Alloway (2013).

All measurements were conducted in triplicate to ensure precision, and quality control was maintained through the use of blanks, duplicates, and certified reference materials.

### Quality assurance and quality control (QA/QC)

Quality assurance and quality control (QA/QC) procedures were applied throughout sample collection, preparation, and laboratory analysis to ensure analytical reliability and data quality. All soil samples were analyzed in duplicate, and procedural blanks were included with each analytical batch to assess potential contamination during sample handling and analysis. Instrument calibration was performed using certified standard solutions, and calibration curves with satisfactory linearity were verified prior to each analytical run.

Limits of detection (LOD) and limits of quantification (LOQ) for each analyzed metal were determined following standard analytical protocols following established analytical detection criteria. Method accuracy was evaluated using recovery tests on spiked samples, which yielded acceptable recovery ranges consistent with international analytical guidelines. Analytical precision was assessed through duplicate measurements, demonstrating good reproducibility of the results.

### Natural attenuation of heavy metals in soil

Natural attenuation of heavy metals in soil refers to the process by which the concentration and toxicity of heavy metals in soil are reduced over time through natural physical, chemical, and biological processes. This process occurs without human intervention and relies on the soil's inherent ability to immobilize, transform, or degrade contaminants. Below is a detailed explanation of the mechanisms involved, along with sources for further reading.

- Dilution and seepage of heavy metals through soil layers
  Heavy metals may migrate vertically through soil layers via seepage and leaching processes, potentially reaching deeper horizons or groundwater systems (USEPA 2006). This process is more pronounced in soils with high permeability and low metal-binding capacity. Soil properties such as pH, clay content, organic matter, and water flow play a critical role in controlling metal mobility. Acidic conditions enhance metal solubility, whereas clay-rich soils and elevated organic matter content promote metal retention and reduce downward migration (Chiaia-Hernández et al. 2022).
- Chemical transformation
  Heavy metals can undergo chemical transformations that alter their mobility and toxicity through redox reactions. For example, chromium may be reduced from the highly mobile and toxic Cr(VI) to the more stable Cr(III), while arsenic may transform into less soluble forms under reducing conditions, leading to partial immobilization in soils (Alloway 2013; Adriano 2001).
- Biological degradation of heavy metals in soil
  Biological processes contribute to natural attenuation through microbial and plant-mediated mechanisms. Certain microorganisms can immobilize or transform heavy metals via bioaccumulation, biosorption, and biotransformation, while plants may uptake metals and reduce soil concentrations. However, excessive metal loads may inhibit biological activity, limiting remediation efficiency (Dixit et al. 2015; González Henao and Ghneim-Herrera 2021).
- Immobilization (sorption and precipitation)
  Heavy metals can be immobilized through sorption onto clay minerals, organic matter, and iron or manganese oxides, reducing their mobility and bioavailability. In addition, metals may precipitate as insoluble hydroxides, carbonates, or sulfides, depending on soil chemistry. Precipitation is strongly pH-dependent and represents a key mechanism for stabilizing Cd, Pb, Zn, Cr, and Ni in contaminated soils (Shacklette & Boerngen 1984; Nriagu 1989; Pacyna et al. 2009)
- Plant uptake (phytostabilization)
  Some plants can absorb heavy metals from the soil and store them in their roots, reducing metal mobility and preventing further spread. This process is part of phytoremediation, a natural or assisted attenuation strategy (Salt et al. 1998; Lasat 2002).
  Natural attenuation is a passive but effective process for reducing the impact of heavy metals in soil over time. It relies on immobilization, chemical transformation, biological activity, and dilution. While it is a sustainable and low-cost approach, its effectiveness depends on site-specific conditions and requires careful monitoring to ensure long-term success.

### Statistical analysis

#### Coefficient of variation (CV) analysis

To assess the seasonal and annual variability of heavy metal concentrations in Mosul soils, the coefficient of variation (CV) was calculated for each metal as the ratio of the standard deviation (SD) to the mean concentration, multiplied by 100 (CV = SD/Mean × 100). This metric provides an indicator of relative variability across sites and between seasons or years. Higher CV values indicate greater variability, reflecting the potential influence of natural processes or anthropogenic activities on metal distribution (Kabata-Pendias 2011; Chen et al. 2015a, b).

#### Principal component analysis (PCA)—modeling section

Principal Component Analysis (PCA) was applied to identify relationships among heavy metals and to infer potential sources of contamination in Mosul soils. Prior to PCA, data were standardized using z-score normalization to eliminate scale effects and ensure comparability among variables with different units, expressed as:

$${PC}_{K}={X}_{a1K1}+{X}_{a2K2}+\dots +{X}_{{a}_{p}}{k}_{p}$$

where X1, X2,…,X are the original metal concentration variables, a _p_ are the loadings**,** indicating the contribution of each metal to the principal component, k = 1,2,…,p (number of PCs).

Data Standardization was applied prior to PCA to ensure comparability of metals with different units or ranges:

$$\mathrm{Zij}=\frac{Xj-Xij}{SDj}$$

where

Zij =: The standardized value of metal j in sample i, so that all variables are on the same scale (mean = 0, SD = 1), Xij is the concentration of metal, X​ is the mean, and SDj is the standard deviation.

The standardized dataset was then used to compute the covariance matrix, from which eigenvalues and eigenvectors were extracted. Each eigenvector defines a principal component, while its corresponding eigenvalue represents the proportion of total variance explained by that component. Components with eigenvalues greater than one were retained, and a scree plot was used to support component selection. Variables with high loadings on the same PC were interpreted as reflecting common controlling factors or potential contamination sources, either natural or anthropogenic (Jolliffe 2002; Zhang et al. 2016).

The suitability of the dataset for PCA was evaluated using the Kaiser–Meyer–Olkin (KMO) measure of sampling adequacy and Bartlett’s test of sphericity. All statistical analyses, including PCA, were conducted using IBM SPSS Statistics (version 26.0).

## Results and discussion

### pH value

Soil pH is a key factor controlling the mobility and bioavailability of heavy metals through its influence on soil chemical equilibria. Across all sites, pH values indicated predominantly neutral to slightly alkaline conditions during both study years (Tables 2, 3, 4). Slightly lower pH values were observed at selected heavily polluted locations (e.g., S3, S4, and S7), which likely reflect the presence of residual acid-forming byproducts associated with past conflict-related activities.

**Table 2:** The mean, minimum, and maximum values of soil samples and heavy metal concentration in all sites during 2022

2022		pH	EC mS/cm	Salinity %	Cd mg/kg	Pb mg/kg	Zn mg/kg	Cr mg/kg	Ni mg/kg
	WHO limits	6.5 − 9.0	0.5–2	3%	0.06	10	50	100	40
	World surface rock	-	-	-	0.2	16	127	71	49
S1	Mean	7.29	0.90	1.31	2.83	21.4	148.5	34.2	40.5
	Max	7.50	0.64	2.10	3.26	25.5	185.9	48.2	55
	Min	7.00	0.46	0.20	1.58	11.9	90.4	23.8	22.9
S2	Mean	7.52	0.92	1.55	3.48	25.8	152.8	49.6	51
	Max	7.90	0.77	2.10	4.07	31.2	191.3	69.7	69.3
	Min	7.20	0.49	0.50	1.98	14.5	93.1	34.3	28.8
S3	Mean	7.50	1.42	2.63	4.87	50.0	348.5	128.2	111
	Max	8.20	2.30	4.00	5.84	62.3	438.3	183.6	150.7
	Min	6.86	1.55	1.38	2.84	29.0	213.2	90.4	62.7
S4	Mean	7.20	1.25	2.55	4.34	46.7	342.0	121.2	101
	Max	7.45	1.99	3.40	5.16	58.1	430.2	171.5	138
	Min	6.93	0.88	1.90	2.51	27.1	209.2	84.5	57.4
S5	Mean	7.24	1.45	2.35	2.45	20.3	159.3	43.0	41.2
	Max	7.50	2.03	3.60	2.81	24.1	262.0	56.3	51.7
	Min	6.93	1.04	1.99	1.39	11.2	31.2	27.7	28.1
S6	Mean	7.41	1.09	2.10	4.12	28.8	308.7	88.8	65.3
	Max	7.89	1.09	3.40	4.89	38.2	388.1	130.0	88.8
	Min	6.98	0.79	0.90	2.38	17.8	188.8	64.0	37.0
S7	Mean	6.96	1.63	2.08	4.77	47.8	325.9	111.9	79.4
	Max	7.40	2.07	3.30	5.63	56.3	404.7	154.1	107.8
	Min	6.67	1.15	1.38	2.77	27.7	199.3	75.9	44.9
S8	Mean	7.41	0.89	2.18	3.80	44.5	263.6	102.2	71
	Max	8.12	1.81	2.90	4.48	55.2	331.1	138.0	82
	Min	6.99	0.67	1.80	2.18	25.7	161.0	68.0	40.3

**Table 3:** The mean, minimum and maximum values of soil samples and heavy metal concentration in all sites during 2023

2023		PH	EC mS/cm	Salinity %	Cd mg/kg	pb mg/kg	Zn mg/kg	Cr mg/kg	Ni mg/kg
	WHO limits	6.5 −9.0	0.5–2.0	3%	0.06	10	50	100	40
	World surface rock	-	-	-	0.2	16	127	71	49
S1	Mean	7.41	0.98	1.40	2.80	18.30	146.53	33.15	34.6
	Max	7.72	1.16	1.56	2.92	24.92	173.86	40.13	38.4
	Min	6.87	0.55	1.2	2.7	15.7	145.7	32.2	30.8
S2	Mean	7.48	0.78	1.49	3.0	25.56	150.28	49.50	46.8
	Max	7.70	0.85	1.63	3.67	27.35	189.62	56.06	51.9
	Min	6.9	0.71	1.3	3.4	23.6	158.9	44.9	41.7
S3	Mean	6.87	2.40	2.29	5.81	48.55	350.63	129.3	108.1
	Max	7.32	2.73	2.56	6.06	51.95	403.25	143.5	148.0
	Min	6.5	1.85	2.0	5.5	44.8	223.0	115.1	96.3
S4	Mean	7.62	1.52	2.58	4.63	44.75	348.35	118.7	101.4
	Max	7.79	1.67	2.82	4.83	47.88	414.91	131.7	112.6
	Min	6.9	1.13	2.3	4.4	41.3	347.8	105.7	90.3
S5	Mean	7.45	1.16	2.57	2.42	18.15	156.28	42.92	34.35
	Max	7.61	1.27	2.88	2.52	21.56	185.26	47.64	38.13
	Min	6.8	1.05	2.3	2.3	18.6	155.3	38.2	30.6
S6	Mean	7.51	1.24	2.40	3.2	30.15	246.78	110	63.0
	Max	7.68	1.66	2.62	4.02	32.26	291.34	122.1	69.9
	Min	6.9	1.13	2.1	3.7	27.8	244.2	97.9	56.1
S7	Mean	7.34	1.67	3.01	4.44	42.40	313.56	102.8	74.1
	Max	7.60	1.89	3.37	4.63	50.72	371.62	121.92	82.3
	Min	6.8	1.10	2.6	4.2	43.8	311.5	97.8	66.0
S8	Mean	7.79	1.23	2.29	3.8	46.55	251.56	98.60	65.62
	Max	7.97	1.92	2.50	4.24	49.92	297.63	118.33	72.84
	Min	7.32	0.93	2.0	3.9	43.1	249.5	94.9	58.4

**Table 4:** Statistical analysis of all values in the city during 2022 and 2023

Element	Year	Mean	Std	Min	Max	25%	50%	75%
pH	2022	7.32	0.19	6.96	7.52	7.23	7.35	7.43
pH	2023	7.43	0.39	6.55	7.79	7.12	7.58	7.52
EC	2022	1.26	0.27	0.90	1.63	1.05	1.33	1.44
EC	2023	1.36	0.53	0.55	2.74	0.97	1.19	1.25
Salinity	2022	2.09	0.46	1.31	2.63	1.94	2.14	2.40
Salinity	2023	2.25	0.55	1.40	3.01	2.09	2.34	2.57
Cd	2022	3.83	0.67	2.45	4.87	3.31	3.96	4.44
Cd	2023	3.78	0.99	2.42	5.81	3.34	3.96	4.48
Pb	2022	33.31	11.98	18.70	47.40	23.10	34.45	43.43
Pb	2023	34.36	14.46	20.15	48.55	24.50	37.45	46.84
Zn	2022	256.16	88.82	148.53	348.48	157.66	286.13	329.9
Zn	2023	245.36	98.70	159.80	403.8	173.28	270.67	348.8
Cr	2022	84.89	32.43	34.16	128.22	47.97	95.50	114.2
Cr	2023	84.24	39.95	36.15	129.30	48.61	108.22	112.1
Ni	2022	69.62	21.00	37.15	150.7	48.36	68.21	84.91
Ni	2023	66.03	29.93	34.35	148.15	43.79	64.31	81

A general tendency toward higher pH values in 2023 suggests progressive soil neutralization processes, potentially driven by rainfall-induced leaching of acidic residues, mineral dissolution, and ion exchange reactions. These patterns are consistent with the behavior of arid-region soils, particularly Aridisols and Calcisols, where carbonate buffering commonly promotes near-neutral to alkaline conditions.

### % Salinity

Soil salinity exhibited clear seasonal variability across the study area (Tables 2, 3, 4). Higher salinity levels were generally observed during dry seasons, reflecting enhanced evaporation and limited leaching under arid climatic conditions, whereas lower values occurred during wet seasons due to dilution and downward percolation of soluble salts.

A slight overall increase in average salinity during 2023 indicates reduced rainfall efficiency in flushing salts from the upper soil layers. Increased variability among sites suggests that local factors such as land use, soil texture, and proximity to pollution sources influenced salt accumulation, particularly in conflict-affected zones. Seasonal salinity fluctuations are therefore an important factor governing the potential mobility and bioavailability of toxic metals in urban soils.

### EC values

Electrical conductivity (EC) is a widely used indicator of soil salinity and overall ionic content and is often applied to assess anthropogenic influences on soil systems, although it does not identify specific dissolved constituents. Variations in EC may result from multiple factors, including salt accumulation, heavy metal deposition, combustion-derived acidic residues, agricultural practices, groundwater interaction, and enhanced evaporation under arid conditions (Benjankar & Kafle 2021).

**Fig. 2:**
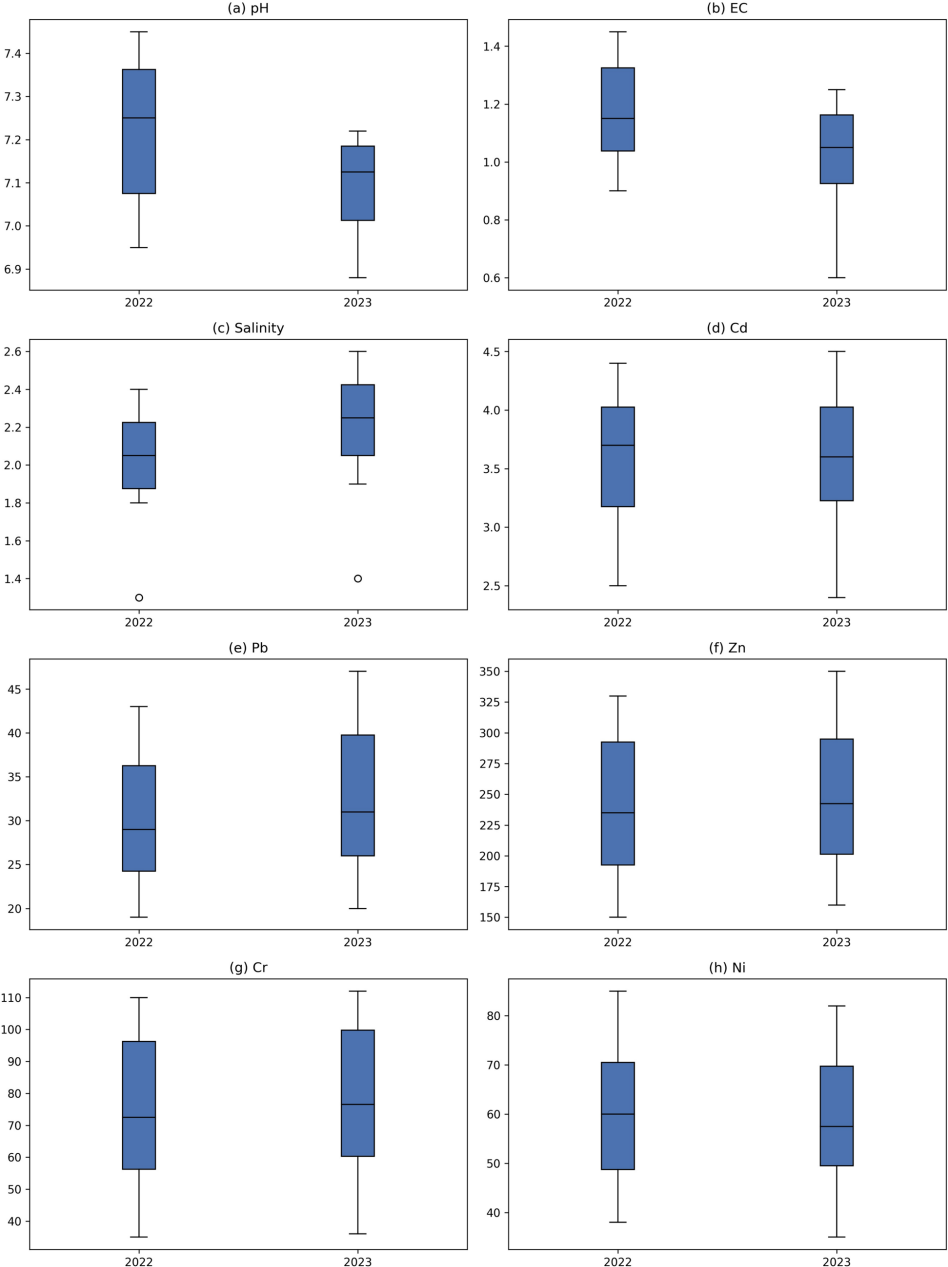
Boxplot distribution of physicochemical parameters and selected heavy metals in 2022 and 2023. Panels represent: (**a**) pH, (**b**) EC, (**c**) salinity, (**d**) Cd, (**e**) Pb, (**f**) Zn, (**g**) Cr, and (**h**) Ni. Plot box of all elements in 2022 vs. 2023

Across the study area, EC values exhibited clear spatial and seasonal variability (Tables 2, 3, 4; Fig. 2). Elevated EC levels were generally associated with conflict-affected sites and agricultural zones, reflecting the combined influence of residual contamination and land-use activities. Higher EC values during dry seasons are primarily attributed to intensified evaporation, which concentrates dissolved salts and ions in the soil matrix, while lower values during wet seasons reflect dilution and leaching effects (Al-Juraisy 2021; Khamidov et al. 2022).

The observed increase in EC can be attributed to several factors, including elevated salinity resulting from higher concentrations of dissolved salts under reduced rainfall conditions, which limits dilution and consequently enhances both salinity and electrical conductivity. In addition, increased evaporation rates promote salt accumulation, further amplifying EC values (Atlas Scientific 2022; Kaushal et al. 2021). The contribution of heavy metals should also be considered, as temporal fluctuations in their concentrations may influence EC measurements, with certain ions exerting a stronger effect on electrical conductivity than others (Mustafa & Ansari 2024).

### Heavy metals

Cadmium (Cd) concentrations exceeded global average soil levels and WHO guideline values at all sampling sites during both study years. Concentrations ranged from 2.45–4.87 mg/kg in 2022 and 2.41–5.80 mg/kg in 2023, with the highest values consistently recorded at site S3, which is located within a conflict-affected zone (Tables 2 and 3; Fig. 2). Mean Cd concentrations remained relatively stable between years, while increased standard deviation and range in 2023 indicate enhanced spatial heterogeneity, reflecting localized increases at sites S3 and S4 and minor declines elsewhere.

Lead (Pb) concentrations similarly exceeded guideline values at several sites, particularly S3, S4, S6, and S8. Overall Pb levels showed a slight increase in 2023, accompanied by greater variability, suggesting uneven site-specific trends rather than uniform accumulation. Zinc (Zn) concentrations exceeded WHO limits at all locations by approximately three to four times, with peak values observed at sites S3 and S4. Mean Zn levels remained broadly stable between years, while increased variability indicates localized enrichment at selected sites.

Chromium (Cr) and nickel (Ni) exhibited comparable spatial patterns, with elevated concentrations predominantly associated with conflict-impacted and industrial locations. Although mean Cr and Ni concentrations showed slight declines in 2023, increased dispersion metrics suggest persistent spatial heterogeneity. Exceedances of guideline values were most frequent at sites S3, S4, and S7, whereas sites S1 and S5 generally remained within permissible limits.

Overall, contamination severity followed the spatial pattern: S3 > S4 > S7 > S6 > S8 > S2 > S5 > S1, highlighting the strong influence of conflict-related disturbance, industrial activity, and traffic density. Slight reductions in average metal concentrations observed in 2023 may reflect limited natural attenuation processes such as leaching and adsorption; however, these effects appear insufficient to offset ongoing inputs from industrial emissions, military residues, agricultural practices, and atmospheric deposition.

Seasonal analysis indicates higher metal concentrations during dry periods compared to wetter seasons, consistent with evaporation-driven concentration and reduced leaching. The absence of a pronounced seasonal contrast in 2023 likely reflects reduced rainfall during that year, limiting dilution and vertical metal migration. These findings underscore the persistence of heavy metal contamination in Mosul soils and the dominant role of site-specific conditions in controlling spatiotemporal variability.

### Spatial and temporal classification of environmental degradation across sites

To enhance the practical applicability of the findings, the sampled locations were categorized based on their overall contamination levels and observed seasonal fluctuations in heavy metal concentrations. This classification aims to identify high-risk zones and support prioritization in soil remediation planning. The classification combined average concentrations of cadmium (Cd), lead (Pb), nickel (Ni), chromium (Cr), and zinc (Zn), along with seasonal percentage variations and their exceedance relative to WHO guideline limits.

Based on cumulative contamination severity over the 2-year period (2022–2023), the study sites were classified into three degradation categories:

- **High degradation:** Sites S3 and S4 exhibited consistently elevated concentrations of all five heavy metals, with pronounced seasonal peaks during the dry periods. Both sites are located within former conflict zones characterized by ongoing human activity, limited vegetation cover, and historical structural damage. These conditions indicate minimal natural attenuation and correspond to the highest potential ecological and human health risks.
- **Moderate degradation:** Sites S6, S7, and S8 showed intermediate contamination levels with partial seasonal variability. Site S6 (industrial), S7 (residential area affected by past explosions), and S8 (agricultural) reflect mixed pollution sources, including conflict-related inputs and ongoing urban emissions, contributing to persistent soil degradation. 
- **Low degradation:** Sites S1, S2, and S5 consistently recorded lower heavy metal concentrations, with only minor seasonal fluctuations. These sites are located in peripheral or non-impacted zones, characterized by higher vegetation cover and reduced human disturbance, suggesting more favorable conditions for natural attenuation.

This zonal classification not only reinforces the spatial heterogeneity of contamination but also provides a foundation for targeted intervention strategies. It is recommended that S3 and S4 be prioritized for immediate remediation, while S6–S8 should be subjected to continued monitoring. S1, S2, and S5 may serve as reference or control sites in future longitudinal studies.

In addition to spatial contrasts, seasonal dynamics further influenced the classification of environmental degradation across sites. Seasonal comparisons indicate that concentrations measured during the dry season (series 2: summer and autumn 2022) were generally higher than those in the rainy season (series 1: winter and spring 2023). This pattern is attributed to rainwater percolation leaching heavy metal precipitates from upper soil layers during wetter periods. The urban soil’s porous nature and mineral matrix low in organic matter reduce adsorptive binding capacity for heavy metals, favoring their dissolution or precipitation during dry spells (Muhaimeed et al. 2014; Mouni et al. 2009). Consequently, rainy conditions promote the migration of metals into deeper soil layers and aquifers (Odat and Ahmed 2011; Oluyemi et al. 2008; Sharma et al. 2020; Altahaan & Dobslaw 2024a, b; Altahaan & Dobslaw 2025b).

A comparison between series 3 (winter–spring 2023) and series 4 (summer–autumn 2023) revealed no clear seasonal differentiation, likely due to the pronounced decline in rainfall during 2023 (Directorate of Transport and Communication Statistics 2022; Altahaan and Dobslaw 2025a, b). Instead, the observed variations were predominantly site-specific, with localized increases and decreases in contamination reflecting heterogeneous responses among individual sites rather than a consistent seasonal pattern across the study area.

### Statistical study

The *t*-test was applied exclusively for paired seasonal comparisons within individual sites. Prior to applying the *t*-test, data normality was assessed using the Shapiro–Wilk test, confirming that the assumptions of parametric testing were satisfied.

#### T test for seasonal variation of all parameters

The *P*-value derived from the statistical *T*-test serves as a threshold to determine the significance of differences between two test series. *P*-values below 0.05 indicate statistically significant differences. In 2022, significant seasonal variations were observed between series 1 and series 2 for heavy metals such as Cd, Pb, Cr, and Ni (*P* < 0.05). Conversely, zinc did not show a significant seasonal effect (*P* > 0.05), likely due to its consistently elevated concentrations across all locations, suggesting that zinc inputs are not primarily associated with military conflict impacts (see Table 5).

**Table 5:** T-test value between series 1 and series 2 of all heavy metals in soil samples in 2022

Heavy metal	*T*-test	*P*	DF*	Status**
pH	−0.27066	0.395299	14	0
EC	−1.71791	0.0242309	14	1
%Sal	−1.39329	0.023361	14	1
Cd	−2.25197	0.020451	14	1
Pb	−1.87422	0.04096	14	1
Zn	−0.85513	0.203439	14	0
Cr	−1.75523	0.045335	14	1
Ni	−1.88613	0.040098	14	1

*DF, degree of freedom; **0 =  not significant, 1 = significant

For 2023, the *T*-test results showed no significant seasonal differences between series 3 and series 4 for the heavy metals tested (Cd, Pb, Zn, Cr, and Ni), with all *P*-values exceeding 0.05 (see Table 6). The absence of significant seasonal variation during 2023 can be attributed to limited precipitation, which restricted natural leaching processes in winter and resulted in reduced seasonal fluctuations in soil properties between winter and summer. This condition, combined with ongoing anthropogenic pollution sources such as vehicle emissions and fuel combustion, likely contributed to the observed stability in soil composition.

**Table 6:** T-test value between series 3 and series 4 of all heavy metals in soil samples in 2023

Heavy metal	*T*-test	*P*	DF*	Status**
pH	4.611	0.0002	14	1
EC	−1.774	0.023	14	1
%Sal	−1.575	0.049	14	1
Cd	−1.265	0.113	14	0
Pb	−0.857	0.203	14	0
Zn	−1.144	0.136	14	0
Cr	−0.448	0.331	14	0
Ni	−0.845	0.206	14	0

*DF, degree of freedom; **0 =  not significant, 1 = significant

Nonetheless, significant seasonal differences (*P* < 0.05) were detected in soil pH, electrical conductivity (EC), and salinity. These changes are attributed to agricultural irrigation and evaporative concentration of dissolved ions (e.g., calcium, magnesium, potassium) during summer. In addition, the use of fertilizers can introduce minerals such as phosphates and nitrates into the soil, further increasing ion concentrations and consequently elevating EC and salinity values (Tables 5 and 6). These results are consistent with findings by Fadl et al. (2024), highlighting the combined effects of climatic variability and human activities on soil geochemistry.

#### Annual variation T-test for all parameters

The annual *T*-test comparing 2022 and 2023 revealed no statistically significant differences, as all *P*-values exceeded the 0.05 threshold. This lack of pronounced annual variation may be linked to reduced rainfall during 2023, in combination with the influence of other pollution sources such as air pollution, as previously discussed (see Table 7).

**Table 7:** T-test value between series in 2022 and series in 2023 of all heavy metals in soil samples

Heavy metal	*T*-test	*P*	DF*	Status**
pH	1.611562	0.064683	30	0
EC	0.542787	0.297906	30	0
%Sal	−0.627685	0.270157	30	0
Cd	−0.139238	0.445623	30	0
Pb	0.165249	0.435555	30	0
Zn	−0.24969	0.403227	30	0
Cr	−0.375585	0.488252	30	0
Ni	−1.95136	0.05020	30	0

*DF, degree of freedom; **0 =  not significant, 1 = significant

The significant seasonal variation observed in 2022 for Cd, Pb, Cr, and Ni (*P* < 0.05) indicates that these metals are sensitive to climate-driven processes such as precipitation and temperature. During rainy seasons, leaching likely transported heavy metals to deeper soil layers, thereby reducing surface concentrations. Conversely, dry seasons promoted metal accumulation at the soil surface due to evaporation and limited dilution.

In contrast, the absence of significant seasonal variation in 2023 across all heavy metals (*P* > 0.05) reflects the unusually low precipitation levels reported by the Directorate of Transport and Communication Statistics (2022). Without sufficient rainfall, natural leaching processes were inhibited, causing soil composition to remain relatively stable between seasons (Altahaan and Dobslaw 2025a, b).

#### Percentage of different for seasonal and annual variation of all parameters in all sites

The seasonal percentage differences for all parameters between series 1 and series 2 (2022) and between series 3 and series 4 (2023) showed notable spatial and temporal variations. The largest salinity fluctuations occurred at sites S1 and S8, with differences of 96.23% and 72.6%, respectively, during 2022 between series 1 and series 2, and 63.1% and 60% in 2023 between series 3 and series 4. These substantial changes are largely attributed to the positioning of S1 and S8 within agricultural zones, where intensified evaporation during summer months likely elevates salt concentrations.

Regarding electrical conductivity (EC), the greatest variation in 2022 was detected at site S3, with a difference of 24.8%. Despite not having the highest salinity, S3’s elevated EC suggests contamination possibly linked to heavy metals, given its location within a conflict-affected area (Heredia et al. 2022). In 2023, the most significant EC difference was recorded at S1, measuring 25.9%. These findings underscore the complex interplay between environmental and anthropogenic influences on water quality parameters over time and across locations (Elrashidi et al. 2015; Ratshiedana et al. 2023).

**Fig. 3:**
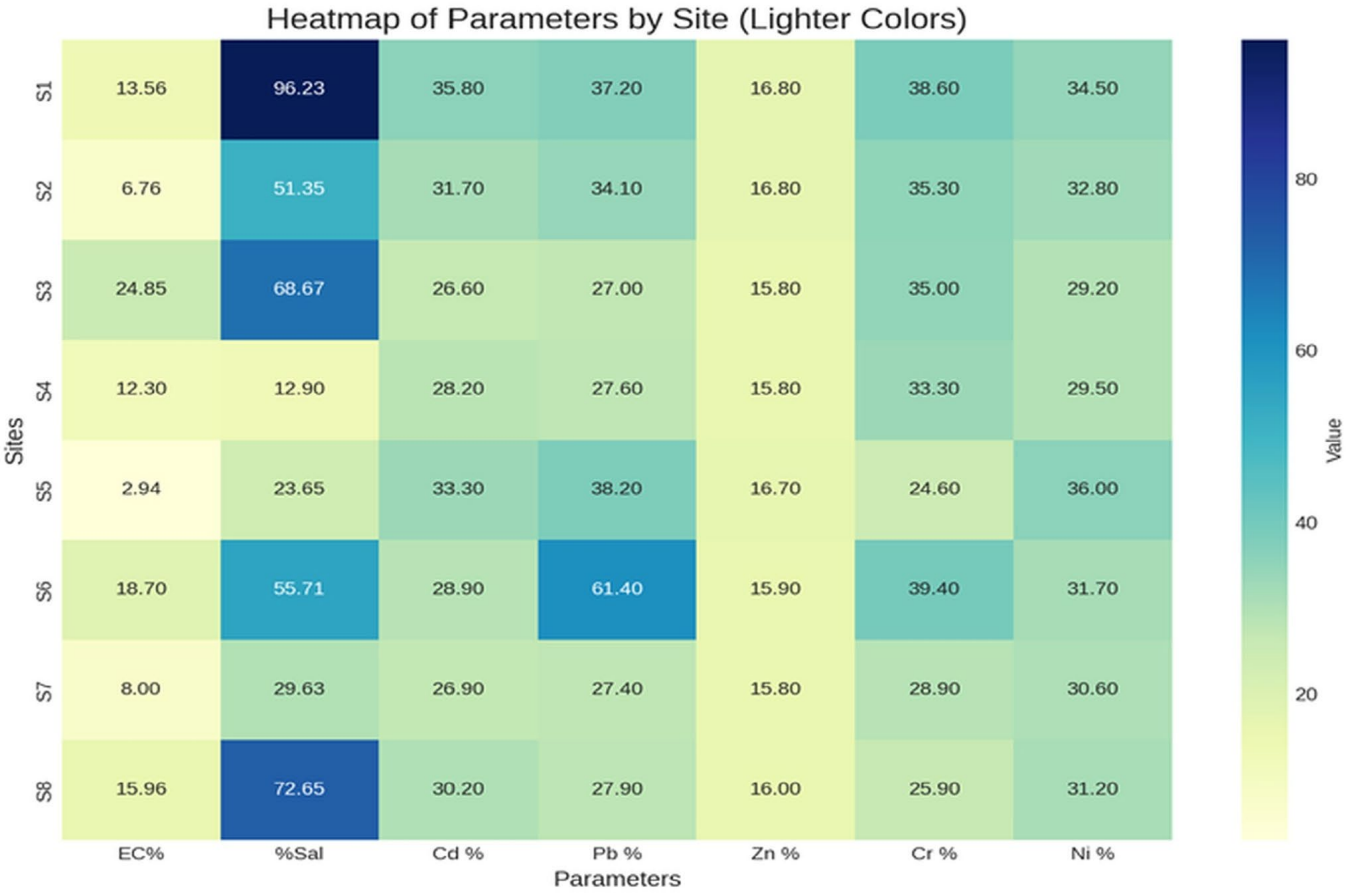
Heat map of the percentage of variation in heavy metal concentration in all sites between series 1 vs. series 2

**Fig. 4:**
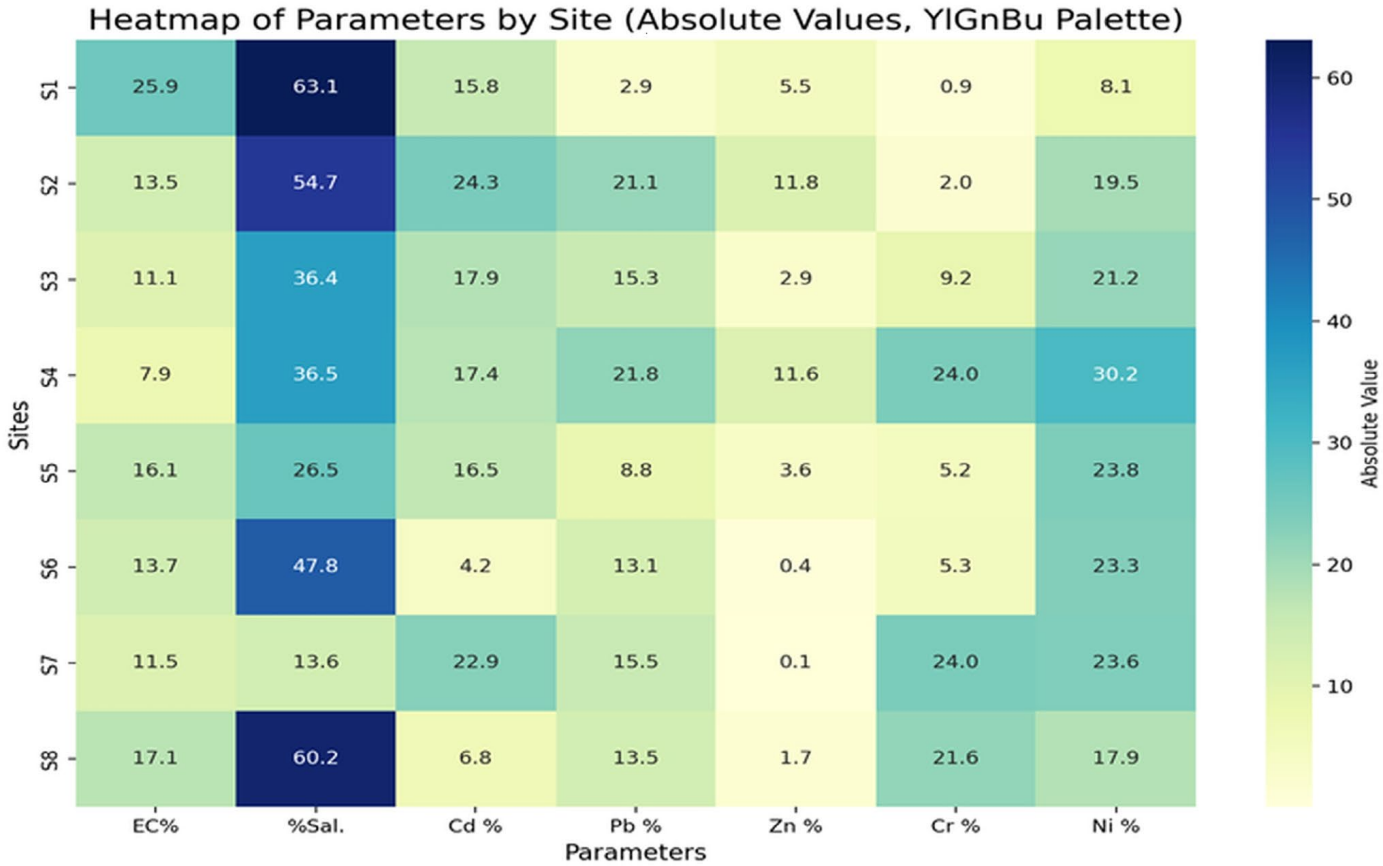
Heat map of the percentage of variation in heavy metal concentration in all sites between series 3 vs. series 4

For heavy metals, seasonal percentage differences in 2022 between series 1 and series 2 varied considerably across sites: cadmium (Cd) ranged from 26.6 to 35.8%, lead (Pb) from 27% to 38.2%, zinc (Zn) from 15.8% to 16.8%, chromium (Cr) from 24.5 to 38.5%, and nickel (Ni) from 29.2 to 35.9%. In contrast, 2023 showed reduced variability between series 3 and series 4, with Cd differences between 6.8 and 24.28%, Pb from 2.9 to 21.8%, Zn from 0.1 to 11.8%, Cr from 0.92 to 24%, and Ni from 8.1 to 30.2%. These patterns are visually depicted in Figs. 3 and 4.

The decreased variability in heavy metal concentrations during 2023 is likely linked to lower precipitation during winter, which reduced soil leaching processes and consequently limited seasonal fluctuations compared to 2022. Annual comparisons between 2022 (series 1 and 2) and 2023 (series 3 and 4) revealed site-specific fluctuations across parameters. EC values rose in 2023 at sites S3, S6, and S7 compared to the previous year, while declining at other locations. Similarly, salinity increased at most sites during 2023.

**Fig. 5:**
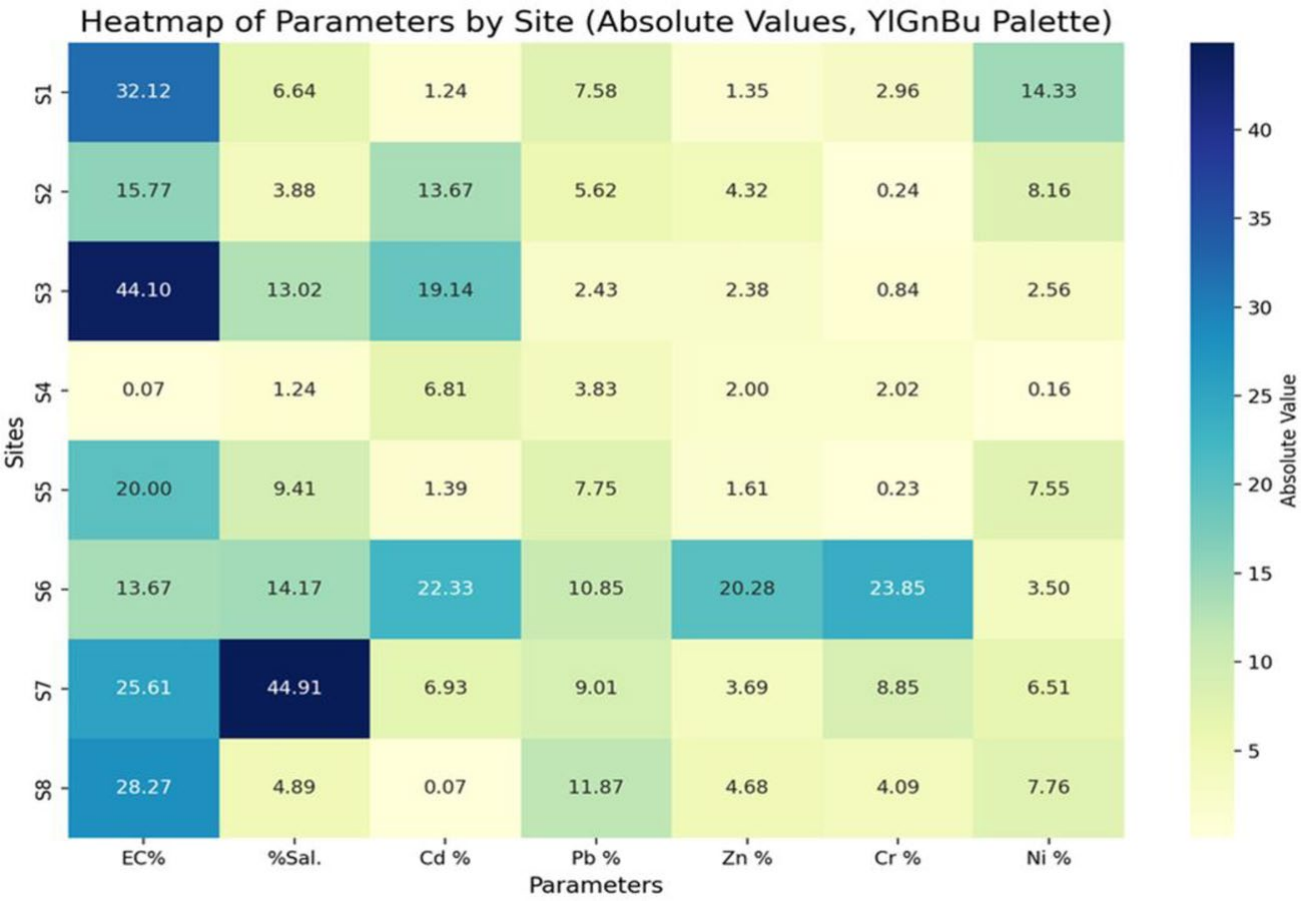
Heat map of the percentage of increase in heavy metal concentration in all sites between 2022 and 2023

Heavy metal concentrations exhibited distinct spatial trends. Cd levels increased at S3, S4, and S8 but declined elsewhere, with percentage changes ranging from 0.07 to 22.4%. Pb concentrations rose at all sites except S1, S5, and S7, with changes between 2.4 and 11.8%. Zn increased at S2 and S4, with differences from 1.35 to 20.2%. Cr levels grew at S3 and S7, ranging from 0.23 to 23.85%, while Ni concentrations decreased across all sites, with differences between 0.16 and 14.3%. These variations are summarized in Fig. 5.

Such spatial and temporal fluctuations are influenced by several factors, including meteorological conditions (e.g., precipitation rates, wind direction, and temperature), soil permeability, and the presence of various pollution sources (Johnston 2023). Notably, pollution from fuel combustion stemming from generators and vehicles has intensified in the post-conflict period, contributing significantly to continued soil contamination (Directorate of Transport and Communication Statistics 2022; Altahaan and Dobslaw 2025a, b).

#### Coefficient of variation (CV) analysis

The calculated coefficient of variation (CV) values showed clear differences in the relative variability of soil parameters between 2022 and 2023. Among the physical and chemical parameters, pH recorded the lowest CV values (2.6% in 2022 and 5.2% in 2023), indicating stable seasonal and annual behavior with minor fluctuations. In contrast, electrical conductivity (EC) and salinity showed higher variability values (21.4% and 22.0% in 2022; 39.0% and 24.4% in 2023, respectively), reflecting greater sensitivity to environmental and anthropogenic influences, such as irrigation methods, climate change, and salt accumulation.

For heavy metals, the variability was more pronounced. Cadmium (Cd) showed moderate CV values (17.5% in 2022 and 26.2% in 2023), indicating temporal instability likely associated with contamination sources from military operations and debris, as well as human and industrial activities, waste deposition, combustion residues, and vehicle exhaust. Lead (Pb), zinc (Zn), chromium (Cr), and nickel (Ni) also showed higher CV values, particularly in 2023, with Pb (42.1%), Zn (40.2%), Cr (47.4%), and Ni (45.3%) experiencing significant fluctuations. This is attributed to climatic factors such as low rainfall. Overall, this suggests strong spatial and seasonal variation in heavy metal distribution, possibly influenced by local pollution hotspots, soil texture fluctuations, and post-war land-use changes. Overall, the climate change coefficient analysis highlights that pH remains relatively stable, while electrical conductivity, salinity, and heavy metals exhibit greater temporal fluctuations, with 2023 showing stronger fluctuations than 2022. These results emphasize the importance of considering seasonal and interannual factors when assessing soil pollution dynamics, see (Table 8).

**Table 8:** Coefficient of variation of all element during study periods

Element	CV% (2022)	CV% (2023)
pH	2.59	5.25
EC	21.43	38.97
Salinity	22.01	24.44
Cd	17.49	26.19
Pb	35.96	42.08
Zn	34.68	40.23
Cr	38.21	47.45
Ni	30.16	45.34

#### Principal component analysis (PCA)—modeling section

**Fig. 6:**
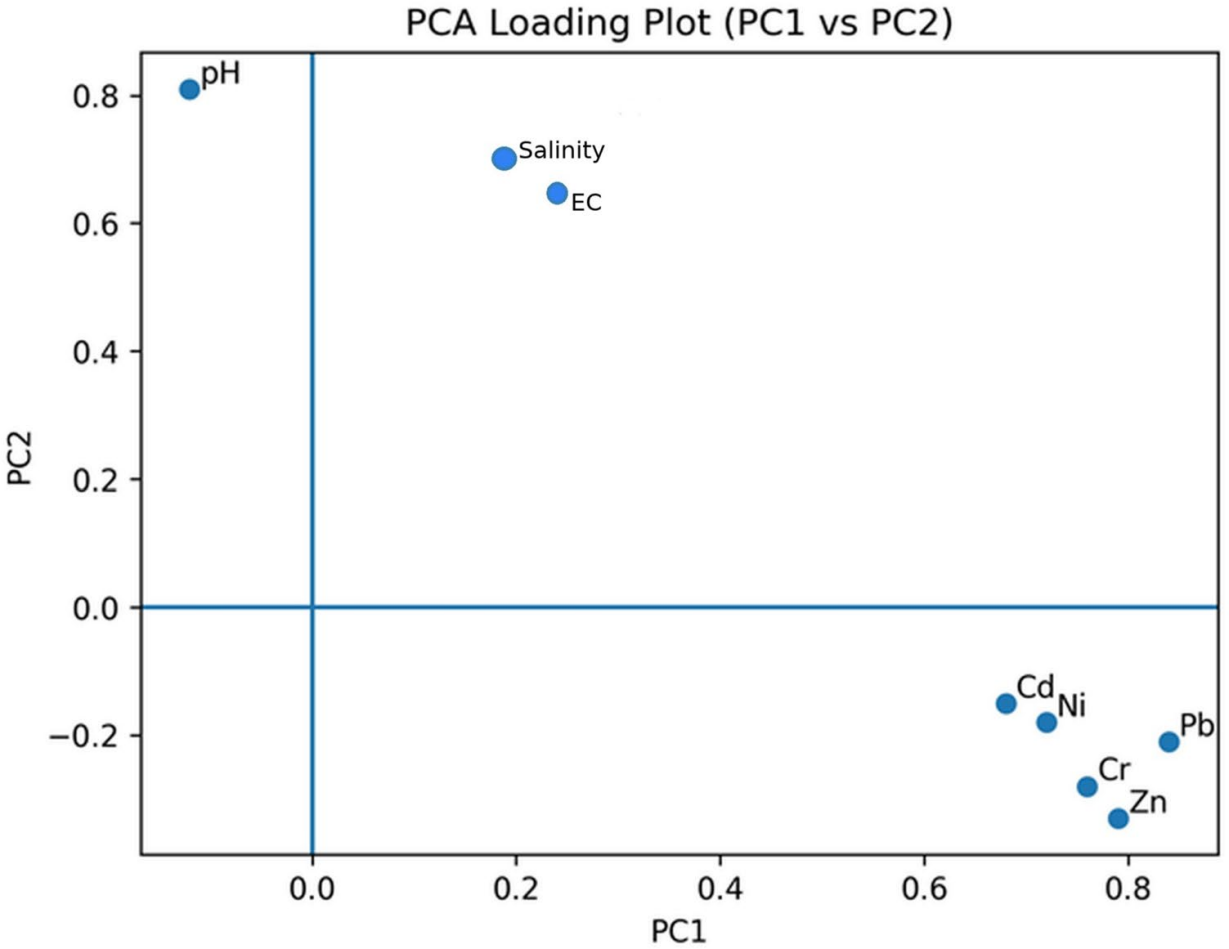
PCA loading plot (PC1 vs PC2) of soil parameters and heavy metals

The Principal Component Analysis (PCA) results revealed that the first two principal components PC1(48.6%) and PC2(21.7%) together explained a substantial proportion of the total variance (about 70%). PC1 showed high positive loadings for Pb, Cd, Zn, Cr, and Ni, indicating that these metals are strongly associated and likely originate from similar anthropogenic sources, such as wartime residues or industrial effluents. In contrast, PC2 was mainly influenced by EC and salinity, suggesting localized geochemical processes or agricultural inputs as potential sources. The separation of variables across the two components highlights distinct spatial and temporal pollution patterns within the sediments, providing a clearer understanding of heavy metal interactions and their environmental implications, see (Table 9; Fig. 6).

**Table 9:** Principal component analysis of all element during study periods

Variable	PC1 loading	PC2 loading
pH	−0.12	0.81
EC	0.35	0.72
Salinity	0.29	0.74
Cd	0.68	−0.15
Pb	0.84	−0.21
Zn	0.79	−0.33
Cr	0.76	−0.28
Ni	0.72	−0.18

#### Spearman correlation coefficient

**Fig. 7:**
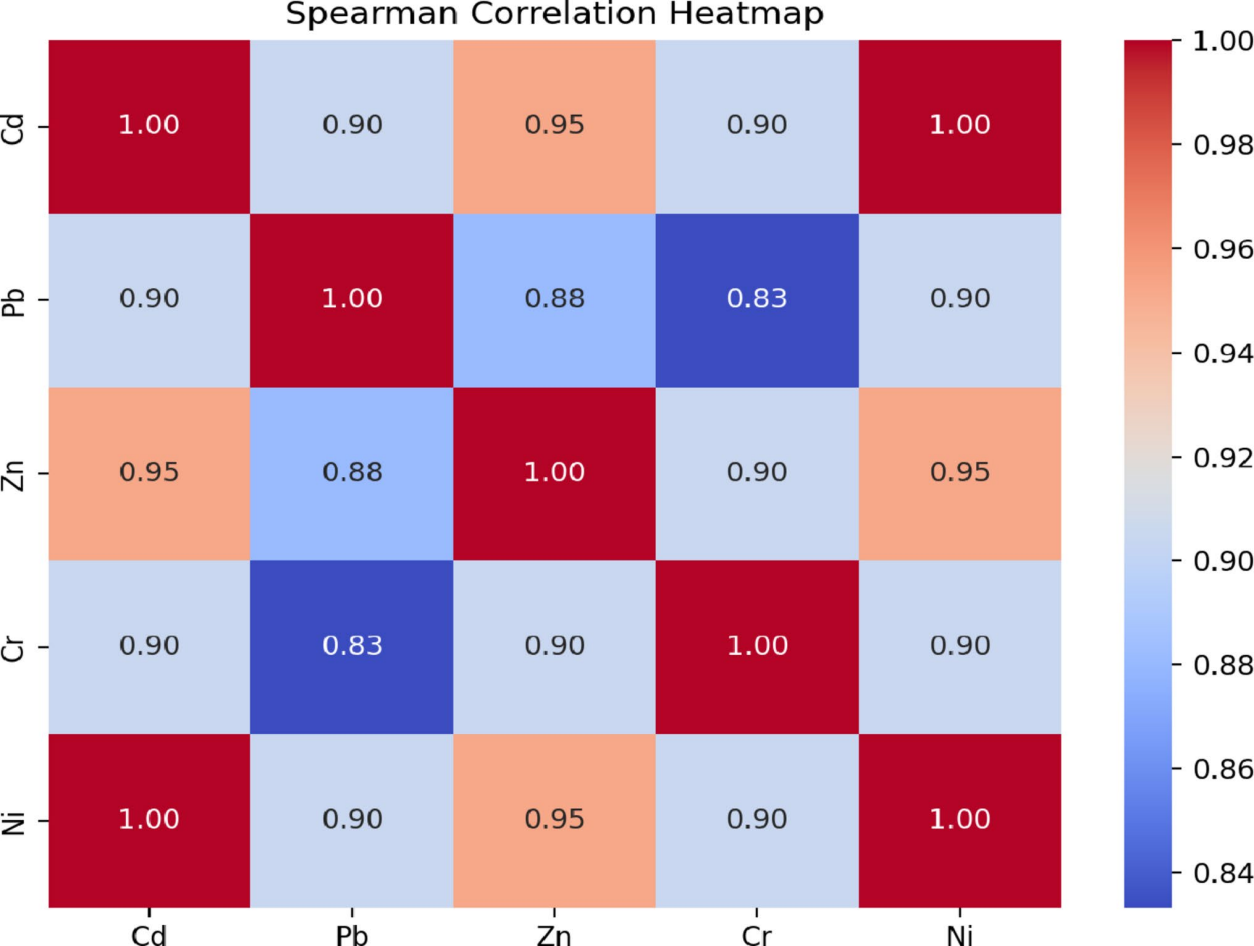
The Spearman correlation matrix and heat map between all elements

The Spearman correlation matrix and accompanying heat map (see Fig. 7) offer valuable insights into the monotonic relationships among the studied parameters. A perfect positive correlation was observed between cadmium (Cd) and nickel (Ni), as well as with zinc (Zn), indicating a strong monotonic association. This suggests these metals might share common sources or exhibit similar environmental behaviors. In addition, lead (Pb) and cadmium demonstrated a robust positive correlation, potentially reflecting their simultaneous presence in specific pollution sources.

Moderate correlations were noted between lead and zinc (0.88) and between lead and chromium (0.83). These slightly weaker associations imply some variability in their origins or environmental dynamics, perhaps due to overlapping but not identical contamination sources.

Overall, the strong correlations observed among most heavy metals suggest they likely derive from shared sources, potentially linked to military activities and conflict-related environmental impacts.

### Climatic influence on temporal dynamics of heavy metal contamination in soil

Climatic variability plays a critical role in shaping the spatial and temporal dynamics of soil contamination, particularly in semi-arid and post-conflict regions such as Mosul. Seasonal patterns in precipitation and temperature directly affect the mobility, dilution, and bioavailability of heavy metals in surface soils. For instance, the rainy seasons (series 1 in 2022 and series 3 in 2023) facilitate the leaching of metals such as Cd, Pb, and Ni into deeper soil layers, as water percolation mobilizes both soluble and weakly adsorbed contaminants. Conversely, during the dry seasons (series 2 and series 4), higher evapotranspiration rates concentrate soluble ions, leading to enhanced surface accumulation of metals and salts.

**Fig. 8:**
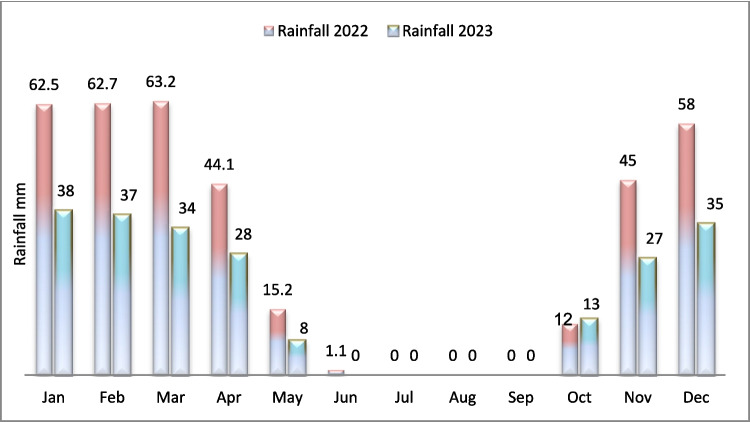
Rainfall rate in Mosul during the years 2022 and 2023 (Altahaan and Dobslaw 2025a)

**Fig. 9:**
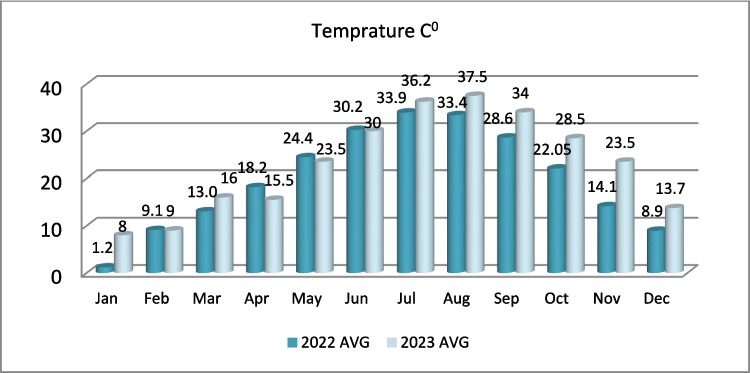
Average temperatures in Mosul during the years 2022 and 2023 (Altahaan and Dobslaw 2025a)

In 2023, the absence of significant seasonal variation was likely due to anomalously low precipitation levels, as confirmed by regional meteorological data. The limited rainfall reduced percolation and leaching effects, causing heavy metal concentrations to remain relatively stable between seasons. This climatic constraint appears to have overridden the natural seasonal variation processes observed in 2022 (Action contre la faim 2022). In addition, elevated temperatures recorded in summer 2023 may have further intensified salt and metal accumulation through enhanced surface evaporation and reduced microbial activity, which would otherwise support metal transformation and degradation (see Fig. 8; Fig. 9).

These findings highlight the sensitivity of soil contamination to climatic inputs and underscore the need to explicitly incorporate meteorological factors into soil pollution assessment models, especially in post-conflict urban environments where environmental recovery is closely linked to climatic conditions.

### Seasonal health risk implications of heavy metal exposure in soil

The seasonal variability in heavy metal concentrations observed in Mosul’s urban soils raises critical concerns regarding public health, particularly in areas with direct human–soil interaction such as residential zones, playgrounds, and agricultural lands. To evaluate potential health implications, a preliminary assessment was conducted using seasonal mean concentrations of cadmium (Cd), lead (Pb), nickel (Ni), chromium (Cr), and zinc (Zn), focusing on differences between wet and dry seasons.

During the dry seasons (series 2 and series 4), elevated concentrations of Cd, Pb, and Ni were consistently recorded across multiple sites, particularly in conflict-impacted zones (S3, S4, and S7). These metals are known to pose chronic health risks through dermal contact, dust inhalation, or consumption of contaminated crops (USEPA 2002; WHO 2017). Cadmium and lead, in particular, have low safe thresholds and are associated with neurotoxic effects and renal dysfunction, especially in children and pregnant women (WHO 2017; Järup 2003).

The lack of significant seasonal decline in 2023 implies a persistent, year-round exposure risk, likely exacerbated by reduced precipitation and ongoing pollutant deposition. This suggests that surface soil contamination remains bioavailable and accessible for human exposure throughout the year, not only during dry periods. The seasonal health risk is further amplified in arid climates, where dust resuspension and human contact with surface soils intensify during hot and dry months.

Although a full quantitative risk assessment (e.g., Chronic Daily Intake or Hazard Quotient) was beyond the scope of this study, the concentration levels observed in summer seasons exceed WHO guideline values for agricultural and residential soil use, indicating a high probability of chronic exposure risks (WHO 2017). These findings underscore the necessity of implementing public health guidelines, such as restricting land use in heavily polluted zones, conducting routine soil monitoring, and promoting phytoremediation and barrier planting strategies (Tangahu et al. 2011; USEPA 2002; Ali et al. 2013).

### Distribution mapping of soil contaminated with heavy metals

Regardless of the difference in soil homogeneity in the region, this assessment was adopted. The spatial classification map is intended to illustrate relative contamination patterns rather than provide an independent quantitative risk assessment. Quantitative pollution indices (e.g., CF and PLI) were comprehensively applied in a previous study to avoid methodological redundancy (Altahaan and Dobslaw 2024c), which was carried out using geostatistical analyses in the ArcGIS 10.4 software (Johnston et al. 2005).

**Fig. 10:**
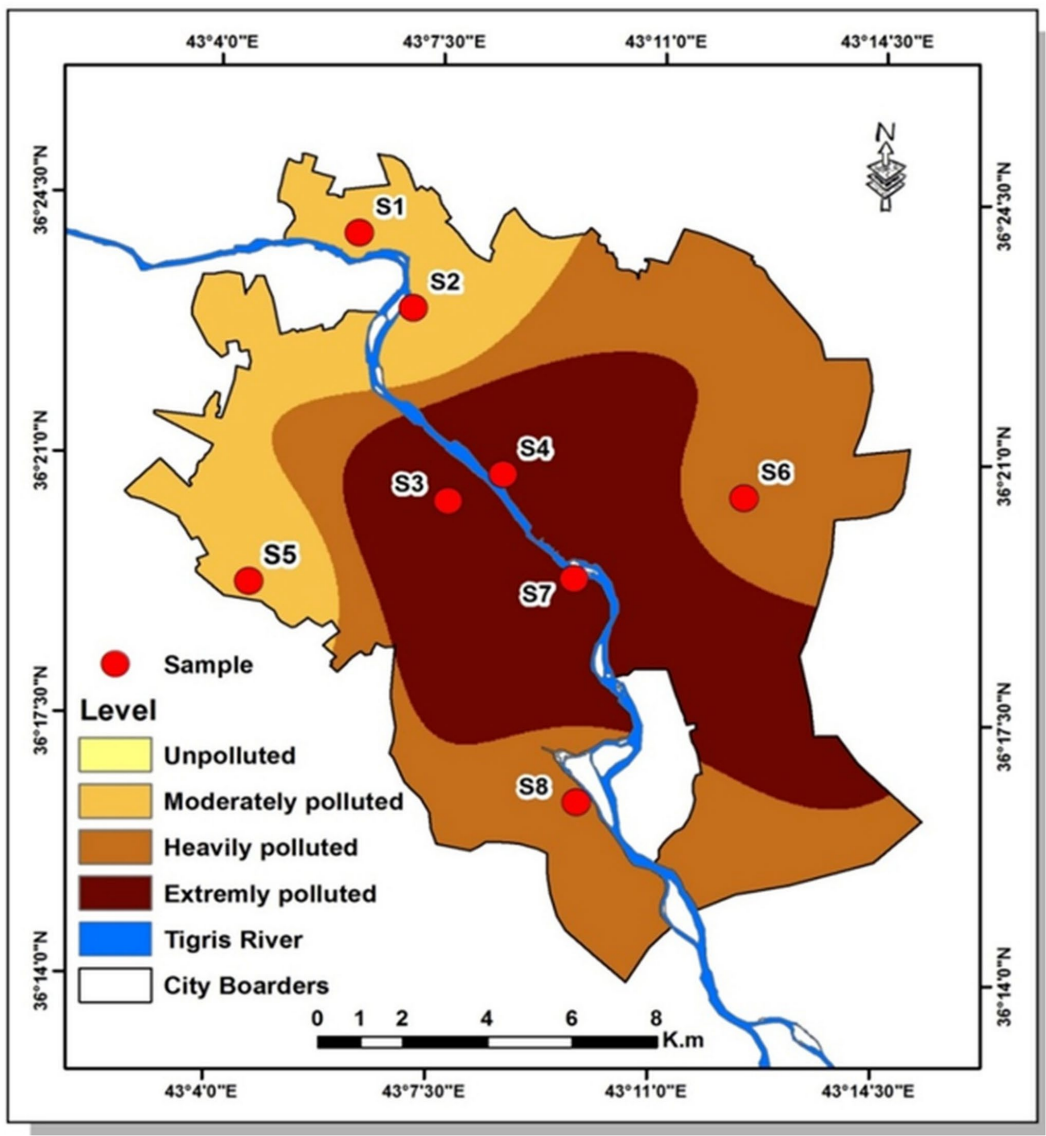
Spatial distribution of heavy metals pollution load index in soil within the urban zone of Mosul

In relation to the total area, the evaluation of the resulting map material shows extreme contamination in 39.47% of the areas (zone with sampling locations S3, S4 and S7 near the conflict zone), severe contamination in 40.09% of the areas (S6, S8), and moderate contamination in 20.43% of the areas (S1, S2, S5). Details can be found in Fig. 10 and Table 10.

**Table 10:** Spatial distribution of heavy metals area (M2) and percentage in the city

Class	Area (M2)	Percentage
Moderate polluted	41.222	20.4352
Heavily polluted	80.8785	40.0936
Extremely polluted	79.623	39.4712

## Global comparison with post-conflict urban environments

Comparable patterns of heavy metal contamination have been reported in other post-conflict urban and peri-urban environments worldwide. In the Gaza Strip, repeated military conflicts and intensive airstrikes have been shown to significantly increase heavy metal concentrations in soils, particularly in agricultural and residential areas, with several metals exceeding background levels and reflecting contributions from military activities and associated emissions (Al-Najar et al. 2015; El-Nahhal 2006; Shomar 2006). These studies emphasize that prolonged conflict and population pressure can lead to persistent soil contamination and pronounced spatial heterogeneity.

Similar observations have been reported in conflict-affected regions of Ukraine, where recent investigations documented elevated heavy metal concentrations in soils following military operations. These increases were associated with explosions, combustion by-products, and damage to industrial and urban infrastructure, resulting in altered soil quality and heightened environmental risk (Yashchenko 2025). Such findings indicate that war-related soil contamination is not limited to arid regions but also affects temperate urban environments.

In comparison with these international case studies, the contamination levels and spatial variability observed in Mosul soils fall within the upper range reported for post-conflict environments. This similarity suggests that the patterns identified in Mosul are representative of a broader global phenomenon linked to armed conflict, infrastructure destruction, and post-war urban recovery. Collectively, these comparisons highlight the long-term environmental legacy of military activities and underline the necessity for targeted remediation measures and sustained monitoring in post-conflict cities.

## Study limitations

This study faced several limitations related to the post-conflict context of Mosul. Access to certain conflict-affected areas was restricted due to security constraints and safety concerns, which limited site accessibility and sampling coverage. In addition, historical baseline data for soil quality, particularly heavy metal measurements, are largely unavailable as a result of war-related damage and the loss of monitoring records, complicating long-term temporal comparisons. The scarcity of prior studies on post-conflict soil contamination in the region further constrained comparative analysis.

Financial limitations and the loss of research funding also restricted the number of collected samples and sampling campaigns, necessitating a focus on seasonal rather than continuous monitoring. Finally, the study addressed total metal concentrations without assessing metal speciation or bioavailability. These constraints should be considered when interpreting the findings and highlight the need for expanded monitoring and future research.

## Conclusion

This study demonstrates pronounced spatial heterogeneity and seasonal variability of heavy metal contamination in the soils of post-conflict Mosul. Elevated metal concentrations were consistently associated with former conflict zones and selected urban–industrial sites, while peripheral and less disturbed areas showed comparatively lower contamination levels, highlighting the strong spatial imprint of land use patterns and conflict intensity.

Natural attenuation processes, including leaching during wet seasons and partial stabilization through soil–metal interactions, appear to contribute to localized reductions in contamination; however, these processes are insufficient to offset ongoing anthropogenic inputs and legacy pollution in heavily affected areas. The spatial contrast between highly degraded and less impacted sites underscores the importance of site-specific management strategies rather than uniform remediation approaches.

From an applied perspective, the findings support the prioritization of immediate remediation in severely contaminated zones, sustained monitoring in moderately affected areas, and the use of low-impact sites as reference locations. The combined application of statistical and geospatial analyses provides a robust and transferable framework for identifying risk zones and guiding soil management strategies in post-conflict urban environments.

## Data Availability

The datasets used and/or analyzed during the current study are available from the corresponding author on reasonable request.
